# The evolution of larger size in high-altitude *Drosophila melanogaster* has a variable genetic architecture

**DOI:** 10.1093/g3journal/jkab454

**Published:** 2022-01-03

**Authors:** Quentin D Sprengelmeyer, Justin B Lack, Dylan T Braun, Matthew J Monette, John E Pool

**Affiliations:** Laboratory of Genetics, University of Wisconsin-Madison, Madison, WI 53706, USA

**Keywords:** adaptive evolution, genetic architecture, size, *Drosophila melanogaster*, quantitative trait locus mapping, local adaptation, genetic differentiation

## Abstract

Important uncertainties persist regarding the genetic architecture of adaptive trait evolution in natural populations, including the number of genetic variants involved, whether they are drawn from standing genetic variation, and whether directional selection drives them to complete fixation. Here, we take advantage of a unique natural population of *Drosophila melanogaster* from the Ethiopian highlands, which has evolved larger body size than any other known population of this species. We apply a bulk segregant quantitative trait locus mapping approach to 4 unique crosses between highland Ethiopian and lowland Zambian populations for both thorax length and wing length. Results indicated a persistently variable genetic basis for these evolved traits (with largely distinct sets of quantitative trait loci for each cross), and at least a moderately polygenic architecture with relatively strong effects present. We complemented these mapping experiments with population genetic analyses of quantitative trait locus regions and gene ontology enrichment analysis, generating strong hypotheses for specific genes and functional processes that may have contributed to these adaptive trait changes. Finally, we find that the genetic architectures indicated by our quantitative trait locus mapping results for size traits mirror those from similar experiments on other recently evolved traits in this species. Collectively, these studies suggest a recurring pattern of polygenic adaptation in this species, in which causative variants do not approach fixation and moderately strong effect loci are present.

## Introduction

Well into the genomic era, considerable debate persists over the types of genetic architectures that underlie adaptive evolution. For example, it is unclear how polygenic adaptive phenotypic changes tend to be—genes of major effect on adaptive traits are often reported (e.g. van’t Hof et al. 2011; Miller et al. 2014), and in the case of local adaptation, these are more likely to overcome the homogenizing force of migration (Yeaman and Whitlock 2011). However, it is possible that most adaptive events may instead involve large numbers of small-effect changes (Pritchard and Di Rienzo 2010; Rockman 2012). It is also unclear how often adaptive variants are selected as newly occurring mutations (e.g. Linnen et al. 2009), vs selection on standing genetic variation after an environmental change (e.g. Colosimo et al. 2005). In the latter case, the detection of “soft sweeps” is a distinct and more challenging exercise than for classic “hard sweeps” (Pennings and Hermisson 2006). It is also unclear how often adaptive variants actually reach fixation, vs remaining polymorphic due to factors such as traits reaching a new optimum or threshold value, changes in selective pressures, balanced equilibria such as heterozygote advantage, or ongoing migration (Stephan 2016; Höllinger et al. 2019; Thornton 2019; Barghi and Schlötterer 2020; Barghi et al. 2020; John and Stephan 2020).

Population genomic scans for natural selection provide some insight into the genetic basis of adaptive evolution, identifying large numbers of loci with signals of recent positive selection, and estimating the frequency at which different functional categories of sites are targeted. However, the biological basis of natural selection at these loci is usually not clear from genetic variation alone, and the properties of adaptive mutations may depend on the biological process (e.g. morphological vs physiological changes; Carroll 2008; Liao et al. 2010). Therefore, an essential complement to population genomic scans is detailed experimental case studies of the genetic basis of specific adaptive phenotypic changes, to gain a clearer and more nuanced understanding of how natural selection operates at the genetic level.

The molecular and evolutionary genetics model *Drosophila melanogaster* provides an efficient system for illuminating the genetic basis of evolutionary change, in part because of its ease of laboratory study, its well-developed molecular genetic toolkit, and its compact and well-annotated genome. *D. melanogaster* expanded from a warm ancestral range in southern-central Africa to occupy diverse worldwide environments (Sprengelmeyer et al. 2020). Latitude and especially altitude gradients allow the comparison of geographically proximate, closely related populations from contrasting environments. Phenotypic differences between genetically similar populations provide ideal raw material for studies of evolution at the genetic level, because the power of population genetic scans for local selection is maximized, and once the relevant genes are identified, the number of plausible causative mutations that differ between populations may be limited.

Size is a fundamental organismal quality. In *D. melanogaster* and other Drosophilids, larger body size is correlated with cooler latitudes (David et al. 1977; Gilchrist and Partridge 1999) and may provide a fitness advantage in cool environments (McCabe and Partridge 1997; Reeve et al. 2000). Instead of a direct effect of size on thermal tolerance (*Drosophila* are small enough to be virtually isothermic with their environment), higher larval density in the tropics may select for earlier pupation, leading to smaller adults, while in cooler regions viability selection may favor larger, more robust adults (Partridge and French 1996).

In other Drosophilid species, larger flies are also found at higher altitudes (Stalker and Carson 1948; Norry et al. 2001), but this phenomenon was not extensively studied in *D. melanogaster* until recently (Louis et al. 1982; Collinge et al. 2006). In the past decade, a unique highland Ethiopian population of *D. melanogaster* was found to be the largest known naturally occurring members of this species, with particularly enlarged wings (Pitchers et al. 2013; Klepsatel et al. 2013; Klepsatel et al. 2014; Fabian et al. 2015; Lack et al. 2016a, 2016b). The increase in wing size is associated with lower wing loading and could benefit flies in highland environments that are persistently cool (limiting the speed of wing movement) and feature thinner air (providing less resistance against fly wings). A plastic decrease in *D. melanogaster* wing loading that occurs at low developmental temperatures is associated with improved flight performance in cold (Frazier et al. 2008). Other studies have found that wing loading does not necessarily predict flight performance under different pressure or temperature conditions (Dillon and Frazier 2006; Hoffmann et al. 2007).

Comparing wing length between a highland Ethiopian population and a low-altitude ancestral range population from Zabmia, phenotypic differentiation (*Q_ST_* = 0.985) greatly exceeded genetic differentiation (genome-wide *F_ST_* = 0.151), implying that directional selection acted on wing length or a pleiotropically correlated trait (Lack et al. 2016a). The species is only estimated to have occupied the Ethiopian highlands about 2,700 years ago (Sprengelmeyer et al. 2020), or roughly 40,000 fly generations ago (based on 15 generations per year; Turelli and Hoffmann 1995; Pool 2015). In light of an effective population size on the order of 1 million for this lineage (Sprengelmeyer et al. 2020), the evolution of larger size has occurred on a recent population genetic time scale (∼0.01 autosomal coalescent units).

There has been some progress on understanding the tradeoffs and mechanisms involved in this population’s size evolution. Compared to a low-altitude Zambian population from the ancestral range, Ethiopian flies lay fewer but larger eggs, which develop into larger adults without prolonging the larval growth phase (Lack et al. 2016b). Ethiopian size changes were found to involve increases in cell size (likely a function of increased somatic ploidy; Smith and Orr-Weaver 1991; Edgar and Orr-Weaver 2001) as well as cell proliferation (Lack et al. 2016b). The evolution of larger wings in Ethiopian *D. melanogaster* was accompanied by a decanalization of wing development, implying that ancestral buffering mechanisms had been disrupted in the course of adaptive trait evolution (Lack et al. 2016a).

The genetic basis of Ethiopian size evolution has not been investigated. Outside Africa, initial progress has been made to identify genes underlying latitude-size clines in *D. melanogaster* outside Africa. In Australia, *Dca* and *srp* are potential contributors to wing and body size differences, respectively (Lee et al. 2011; Chen et al. 2012). Functional experiments (Carreira et al. 2009), association testing (Jumbo-Lucioni et al. 2010; Lafuente et al. 2018; Watanabe and Riddle 2021), and experimental evolution (Turner et al. 2011) have suggested that many genes could influence within-population body size variation. However, quantitative trait locus (QTL) mapping of size differences between high- and low-latitude populations has suggested a few major loci, with uneven chromosomal contributions not predicted by a highly polygenic model (Calboli et al. 2003). Hence, the polygenicity of body size variation may depend on whether diversity is examined within populations where stabilizing selection may predominate, or between populations where adaptive phenotypic evolution is suspected.

In this study, we aim to understand the genetic architecture of adaptive trait evolution, using the Ethiopian population’s thorax and wing size changes as model traits. Here, thorax length represents a proxy for overall body size, whereas wing length represents a trait that has particularly evolved in this population (Lack et al. 2016a). We focus on the polygenicity of trait evolution and genetic predictability within a population. We perform bulk segregant analysis to ascertain QTLs that are involved in thorax and wing size trait evolution. We also use population genetic statistics and gene ontology (GO) enrichment to find the evidence of local adaptation and to identify candidate genes for future functional investigation.

## Materials and methods

### Bulk segregant analysis

Here, we sought to map the larger thorax and wing size of a high-altitude Ethiopian population (from Fiche, Ethiopia, “EF,” 9.81°N, 38.63°E, alt. 3,070 m) compared to a low-altitude ancestral range population (Siavonga, Zambia, “ZI,” 16.54°S, 28.72°E, alt. 530 m). Four independent crosses were conducted, each involving a unique pair of EF and ZI strains: EF8N × ZI403N, EF15N × ZI366N, EF73N × ZI418N, and EF86N × ZI274N. These lines had been inbred for 8 generations from wild-caught isofemale lines, though inbred lines still contain significant levels of residual heterozygosity (Lack et al. 2016c). These lines had been found to be homozygous for common inversions—*In(1)A*, *In(1)Be*, *In(2L)t*, *In(2R)NS*, *In(3L)OK*, *In(3L)P*, *In(3R)K*, *In(3R)Mo*, and *In(3R)P—*by a combination of genomic data analysis (Lack et al. 2016c) and PCR (Lack et al. 2016a). All flies used were raised at 20°C on medium prepared in batches of 4.5 l water, 500 ml cornmeal, 500 ml molasses, 200 ml yeast, 54 g agar, 20 ml propionic acid, and 45 ml tegosept 10% (in 95% ethanol), at room temperature.

**Fig. 1.**
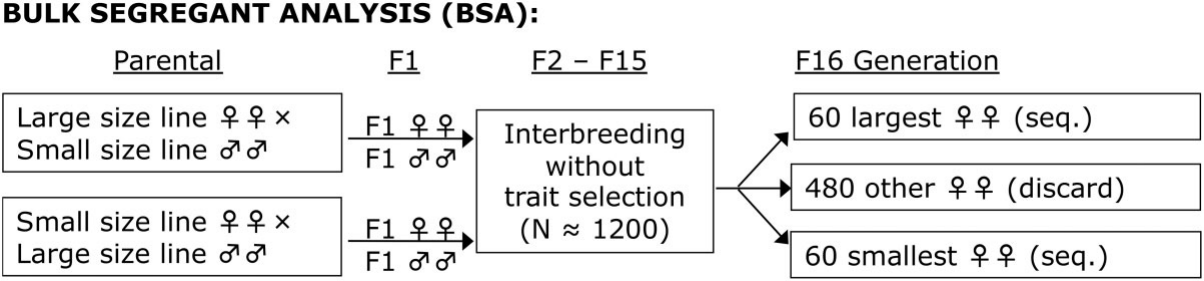
The bulk QTL mapping experimental design is illustrated. As further described in the *Materials and Methods*, F1 offspring of reciprocal crosses were allowed to interbreed in a relatively large population without selection until the F15 generation, at which point 600 females were sorted to obtain the top and bottom 10% for a size trait for sequencing. This design allows a large number of unique recombination events to take place, which should improve mapping performance.

To determine which regions of the genome harbor the causative variants responsible for the evolution of larger thorax and wing size, bulk segregant analysis was performed to detect QTL. Four different population cages were started—1 for each of the Ethiopia-Zambia crosses mentioned. Each population cage is 28 cm × 14 cm × 15 cm and has 14 vials containing the above medium. In each population cage, reciprocal crosses were established between 8 inbred parental individuals of each strain (Zambia and Ethiopia). From each reciprocal cross, 125 F1 offspring of each sex were used to establish the second generation. For the duration of the experiment, nonoverlapping generations were maintained at ∼1,200 individuals (Fig. 1). Adult flies were allowed to lay eggs on the food for 1 week before being removed. The food vials were replaced when adult flies in the cage were 7–10 days old. At the 16th generation, 600 3–5-day-old female flies from each population cage were measured as described below. For each trait, thorax size and wing size, the flies were placed into pools constituting the 10% smallest (*N* = 60) and 10% largest (*N* = 60) individuals, with the remaining individuals discarded. The QTL detection power of this experimental design is expected to be higher for QTLs explaining more than 15% of the parental strain trait difference (Pool 2016—Fig. 7A).

### Body size

To measure thorax and wing size, we followed the protocol described in Lack et al. (2016a). Thorax size measurements were in 3–5-day-old adult females. From each mapping cross, females were photographed with a digital camera attached to a stereo dissecting microscope (AmScope SM-4BX), and thorax length was measured from the base of the anterior humeral bristle to the posterior tip of the scutellum (see Lack et al. 2016b, Fig. A2B). For wing size, we also examined 3–5-day-old adult females from each of the mapping crosses. For 5 females per cross, a wing was removed and photographed at 509 magnification using a digital camera attached to a compound microscope (Olympus BH-2). The length and depth of each wing were then measured using ImageJ version 1.48 (http://imagej.nih.gov/ij/; last accessed January 5, 2022), and we measured a straight line drawn from the intersection of the anterior crossvein and L4 longitudinal vein, to where the L3 longitudinal vein intersects the wing margin (see Lack et al. 2016b, Fig. A2A). For depth, we measured a straight line from the intersection of the L5 longitudinal vein and the posterior wing margin, passing through the intersection of the posterior crossvein and L4, and terminating at the anterior wing margin. For wing area, we imaged individual wings using the “wing grabber” apparatus described by Houle et al. (2003), wing area was determined by outlining each wing using ImageJ version 1.48 (http://imagej.nih.gov/ij/; last accessed January 5, 2022), and the reported area for each cross is the mean of the 5 wings.

### Genome preparation

We sequenced the genomes of pooled samples (*N* = 30 individuals) for the parental lines and 2 such pools for each of the large- and small-size groups (0–5% and 5–10% extremes for each direction, summing to *N* = 60 total for each extreme). Genomic DNA was obtained using a chloroform extraction and ethanol precipitation protocol. The DNA was fragmented with a Bioruptor sonicator (Diagenode), and paired-end libraries with ∼300-bp inserts prepared using NEBNext DNA Library Prep Reagent Set for Illumina (New England Biolabs no. E6000L). Each library’s concentration and quality was analyzed with an Agilent 2100 Bioanalyzer (Agilent Technologies, Inc.). The prepared libraries were sequenced at UW-Madison Biotechnology Center on the Illumina HiSeq 2000 platform. Having concluded that the full 10% extremes would best be analyzed together (Pool 2016), we merged reads from the 0–5% and 5–10% pools (similar numbers of reads were obtained from these pools in each case) before proceeding with the analysis.

### Genome alignment

All the raw data that passed the Illumina filters were processed using a Perl-scripted pipeline. Reads from each sequenced genome were mapped to the *D. melanogaster* reference genome (release 5.57) obtained from Flybase (www.flybase.org), with the default parameters in BWA ver. 0.6.2-r126 (Li and Durbin 2009). Using Stampy ver. 1.0.21(Lunter and Goodson 2011), the BAM files were then remapped. With samtools ver. 0.1.18 (Li et al. 2009), reads were filtered for a mapping quality of 20 and for proper pairs. The BAM files were further processed by removing unmapped reads and sorted by coordinate, and PCR duplicates were marked using Picard ver. 1.109 (http://picard.sourceforge.net; last accessed January 5, 2022). To improve the alignment around indels, we used GATK ver. 3.2 (McKenna et al. 2010). The average depth of coverage per genome was calculated for the parental lines and the low- and high-tolerant lines (Supplementary Table 1).

### QTL mapping

Synchronized mpileup files for the aligned genomes were created with the PoPoolation2 ver. 1.201 software package (Kofler et al. 2011). The 2 large (and 2 small) pools from a given cross were then combined with a custom perl script. Ancestry difference (*ad*) was then calculated with each biallelic SNP (Bastide et al. 2016). Ancestry difference estimates the difference between the proportion of the large-fly pool’s sequencing reads carrying an allele from the large (Ethiopia) parental line and that same proportion from the small-fly pool. It was estimated as:

(1) $$a_{d}=\left(f_{L}{}_{}–f_{S}\right)/\left(p_{L}–p_{S}\right)$$

where *p_L_* is the frequency of the major allele in the large parent, *p_S_* is the small parental allele, *f_L_* is the frequency of the large parent allele in the large pool of F16 offspring, and *f_S_* is that same allele’s frequency in small F16 offspring. The 5 chromosomal arms (X, 2L, 2R, 3L, and 3R) were divided into windows based on SNP density (Lack et al. 2015) which created 2,728, 3,131, 2,357, 2,956, and 2,935 windows, respectively, each roughly 8.4 kb in size on average. Only sites that had a parental strain frequency difference of ≥0.25 were used in the analysis, to avoid noisy ancestry proportion estimates from SNPs with modest frequency differences between parental strains. A simulation-based inference for BSA mapping (SIBSAM) was performed (Pool 2016) to identify significant QTLs and calculate their confidence intervals and effect sizes. SIBSAM is able to evaluate both primary QTL peaks and flanking secondary QTL peaks, evaluating whether ragged peaks contain significant evidence for more than 1 QTL. Forward simulations incorporate recombination in multiple individuals for multiple generations, selection on phenotype in the final generation with additive gene effects, plus environmental variance, and then the sampling of sequence reads to obtain *ad*.

### Genetic differentiation and GO enrichment analysis

QTLs identified in the previous step will contain many genes that may or may not be involved in the evolution of these traits. To help identify the causative genes within the significant QTLs for thorax and wing size, window *F_ST_* and maximum SNP *F_ST_* per window (“SNP *F_ST_*”), and the haplotype statistic *χ_MD_* (Lange and Pool 2016) were analyzed. Genomes from Zambia (*n* = 197) and Ethiopia (*n* = 68) were used from the *Drosophila* Genome Nexus (Lack et al. 2015). The 2 *F_ST_* statistics, quantifying allele frequency differences between populations, were used because window *F_ST_* should be sensitive to locally adaptive selection sweeps with larger linkage effects such as hard sweeps, while SNP *F_ST_* should be sensitive to narrower sweep signals such as those associated with selection on standing variation that result in soft sweeps (Pennings and Hermisson 2006). The *χ_MD_* compares length of identical haplotype blocks among individuals in one population vs another.

For our local adaptation genome-wide scans, each of the 5 chromosomal arms (X, 2L, 2R, 3L, and 3R) were divided into windows based on SNP density (Lack et al. 2015), which created 2,728, 3,131, 2,357, 2,956, and 2,935 windows, respectively, each roughly 8.4 kb in size on average. To narrow down potential candidate genes, a chromosomal arm quantile outlier approach was used to identify genes with an extreme population genetic signal. For each statistic and each chromosome arm separately, we defined a window’s quantile as the proportion of all windows on that chromosome arm with an equal or greater value for that statistic. We classified windows that were in the top 2.5% quantile for any of the 3 statistics as outlier windows. We then grouped neighboring outlier windows together into outlier regions, since they may reflect the same instance of local adaptation. To form an outlier region, a maximum of 2 nonoutlier windows were allowed between 2 outlier windows. Genes associated with outlier windows (overlapping them or the nearest gene in either direction) were retained for subsequent analysis.

We performed a GO enrichment analysis to identify potential functional categories that may contribute to the contrasting phenotypes found between the Zambia and Ethiopia populations. The outlier genes that were identified in the significant QTL regions were used for window-based GO enrichment analysis (Pool et al. 2012). A GO enrichment analysis was conducted for both thorax and wing size. A *P*-value was calculated based on the probability of observing a given number of outlier genes from a GO category. *P*-values were obtained from permutation in which outlier regions were randomly reassigned 10,000 times.

## Results

### QTL mapping

We used bulk segregant analysis to perform QTL mapping for both thorax and wing length using 4 different unique between-population crosses. Each mapping population used individual inbred strains from an ancestral range Zambia population with smaller thorax and wing length, and from the high-altitude Ethiopia population that has evolved larger thorax and wing length. In our bulk segregant analysis, offspring of reciprocal crosses were allowed to interbreed for 16 nonoverlapping generations without selection at a large population size (*N* ≈ 1,200). After the 16th generation, 600 adult females were measured for both thorax and wing length and the top and bottom 10% of individuals were grouped for pooled genomic sequencing (Fig. 1; *Materials and Methods*). SIBSAM (Pool 2016) was then used to identify primary and secondary QTL peaks, along with their estimated effect sizes and genomic confidence intervals.

###  

**Fig. 2.**
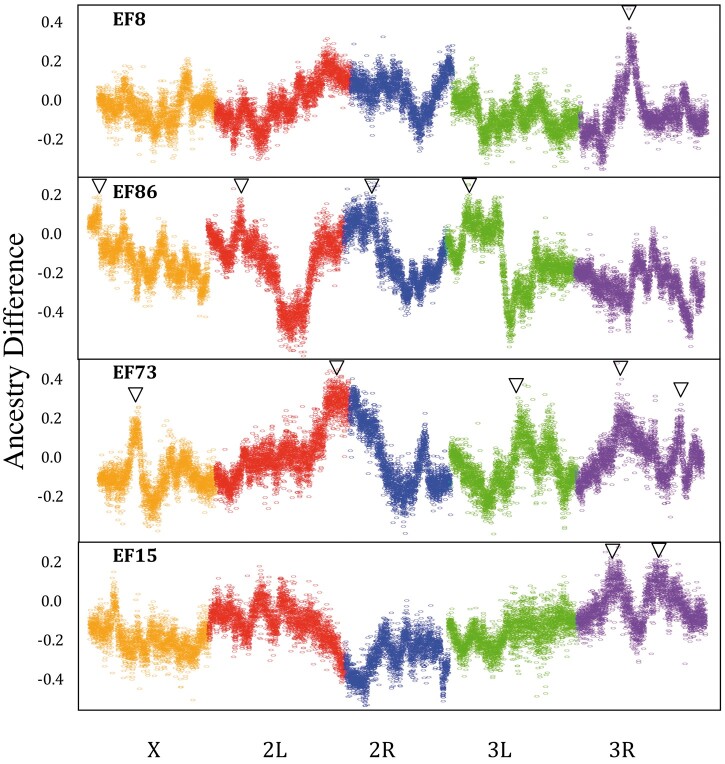
Significant QTL peaks for 4 Ethiopia/Zambia thorax length crosses. A point for each ∼8-kb window corresponds to the average difference in ancestry from the larger parental strain between the large and small F16 pools (*y*-axis). Significant primary or secondary QTL peaks are denoted with an arrow. The significance threshold for primary peaks is approximately 0.17.

For thorax length, 4 Ethiopia × Zambia mapping crosses revealed a total of 12 significant peaks (Fig. 2 and Supplementary Table 2). The EF8N cross had 1 significant peak with an estimated effect size of ∼17%. EF15N had 2 significant peaks, each having an estimated effect size of ∼15%. EF73N had the most significant peaks with a total of 5, and these had estimated effect sizes that ranged between 12% and 20%. EF86N had 4 significant peaks with estimated effect sizes between 13% and 16%.

**Fig. 3.**
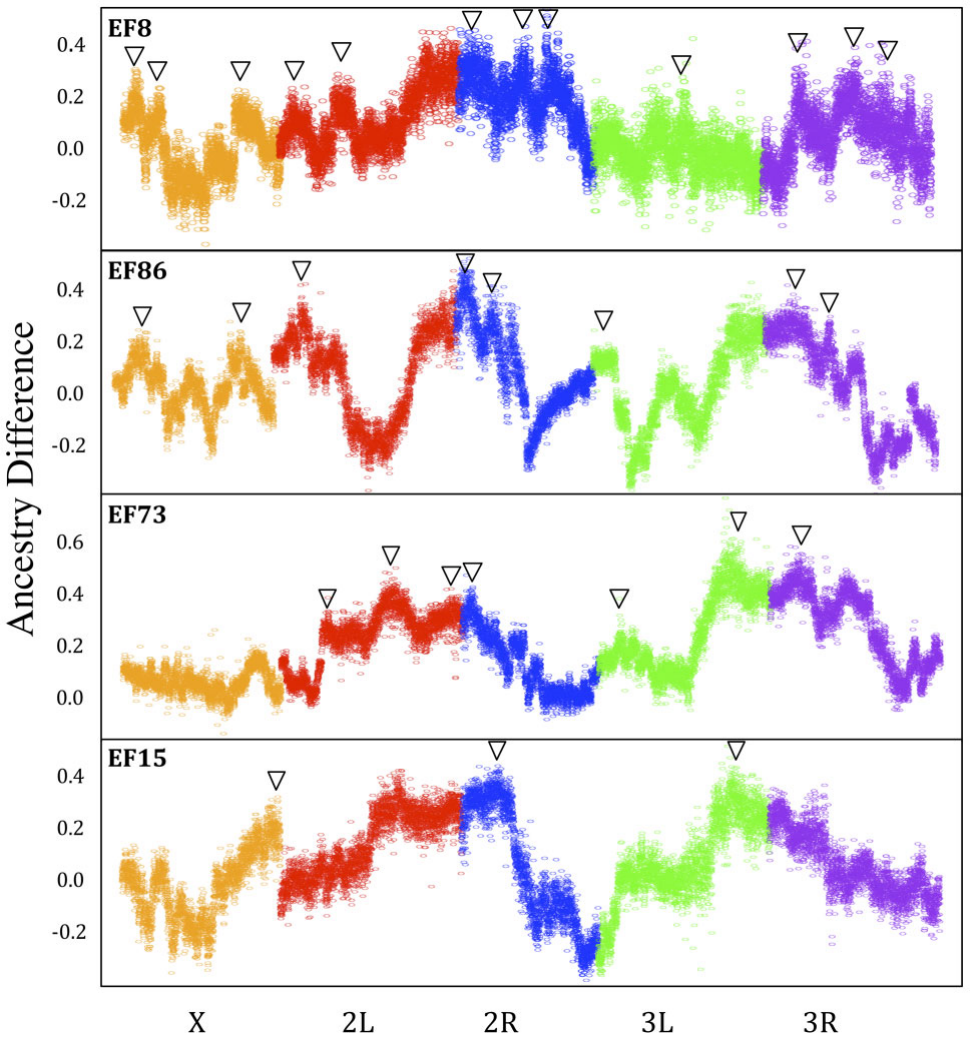
Significant QTL peaks for 4 Ethiopia/Zambia wing length crosses. A point for each ∼8-kb window corresponds to the average difference in ancestry from the larger parental strain between the large and small F16 pools (*y*-axis). Significant primary or secondary QTL peaks are denoted with an arrow. The significance threshold for primary peaks is approximately 0.17.

For wing length, these same 4 crosses revealed a total of 33 significant peaks (Fig. 3 and Supplementary Table 3). EF8N had a total of 12 significant peaks, with estimated effect sizes that ranged between 7% and 24%. EF15N had 3 significant peaks, with estimated effect sizes that range between 16% and 24%. EF73N had a total of 10 significant peaks, with estimated effect sizes that ranged between 6% and 27%. EF86N had 8 significant peaks, with estimated effect sizes that ranged between 11% and 25%.

**Fig. 4.**
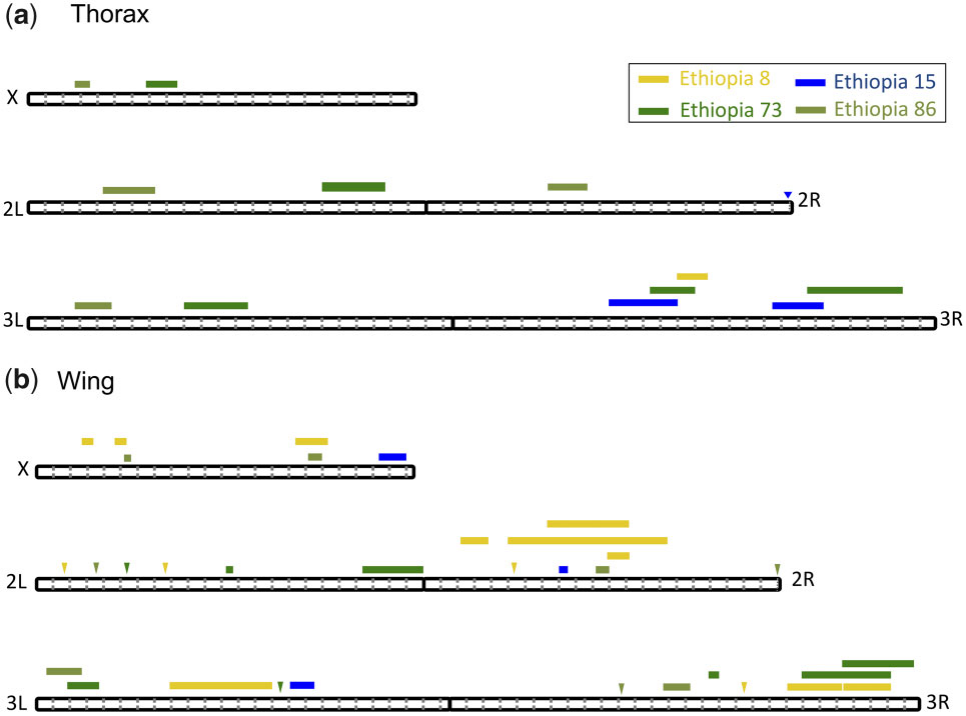
The locations of significant QTLs on the 5 euchromatic chromosome arms of *D. melanogaster*. The colors indicate for 4 Ethiopia strains used in mapping crosses for a) thorax length and b) wing length. The width of each box indicates the 90% CI of each QTL. Intervals that are less than 10 kb in width are marked with triangles. Dotted gray lines indicate Mb increments.

In general, very different QTL landscapes were observed between independent Ethiopia/Zambia crosses (Fig. 4). In some cases, QTLs do overlap between crosses, which may reflect either chance (different QTLs located close together) or else genuine sharing of causative variants underlying thorax and/or wing size. We identified QTL overlap when the QTL peak of 1 cross overlaps with the genomic confidence interval of another cross. For thorax length there are no regions between the 4 Ethiopia crosses where a QTL peak overlapped with another peak’s genomic confidence interval (Fig. 4). However, for wing length between the 4 crosses, there were 12 regions where QTL peaks overlapped with genomic confidence intervals involving 12 of the 33 QTLs (Fig. 4). Within these overlapping peaks, there are no overlap between all 4 crosses.

Some differences in the significant QTLs between crosses could represent chance detection of a shared QTL in some crosses but not others. However, with this experimental design, we expect to have >90% power to detect a QTL with 20% effect size (Pool 2016). Hence, at least for several of the strongest of the QTLs detected here, their absence in other crosses is likely to reflect real differences in genetic architecture.

### Potential targets of local adaptation within QTL regions

Regions of the genome where the Zambia and Ethiopia populations greatly differ in their genetic variation may harbor genes involved in these adaptive traits. We used 3 population genetic statistics, window *F_ST_*, maximum SNP *F_ST_* within a window, and the haplotype statistic *χ_MD_* to identify possible candidate genes for body and wing size evolution within the significant QTLs. Using 3 different statistics is advantageous due to the differing power each statistic has in detecting local adaptation, depending on whether selective sweeps are complete or incomplete, or hard vs soft (Lange and Pool 2016). A quantile approach was used to identify population genetic outlier regions within QTLs that had one of the 3 statistics with a quantile of below 0.025. These local adaptation candidate regions are typically much narrower than QTLs, and hence, there can be multiple outlier regions per QTL, but most outlier regions are associated with just a few genes (Supplementary Tables 4 and 5). There are many genes within these outlier regions with no known role in either thorax or wing size. However, there are also genes known to be involved in size regulation.

**Fig. 5.**
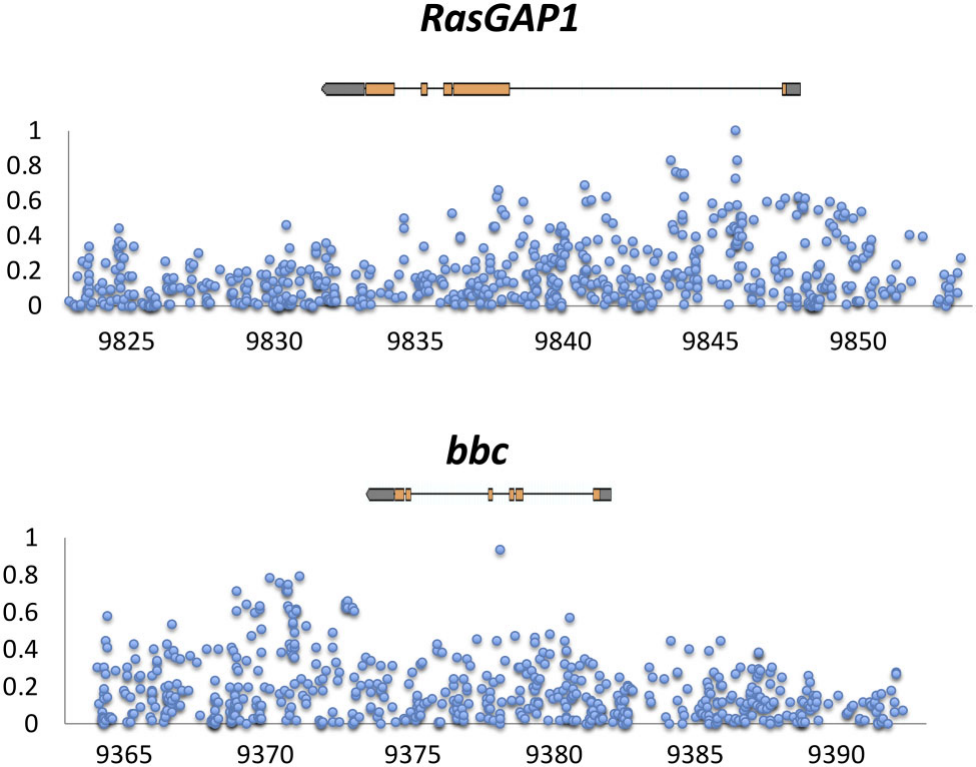
At 2 candidate genes identified for thorax length evolution (*bbc* and *RasGAP1*), small numbers of SNPs show the highest *F_ST_* values between Ethiopia and Zambia. Depicted above is the gene transcript, while the *x*-axis indicates kb position along the relevant chromosome arm (release 5).

For thorax length, genes corresponding to QTLs and population genetic outliers that are known to be involved in growth included *bbc* (Liu et al. 2014), *ct* (Thumm and Kadowaki 2001), *msn* (Kadrmas et al. 2004; Carreira et al. 2009), *RasGAP1* (Dworkin and Gibson 2006), *scyl* (Reiling and Hafen 2004), *spi* (Nagaraj et al. 1999), *srp* (Bánréti et al. 2012; Chen et al. 2012), and *tara* (Bejarano et al. 2008). Of these, *bbc* and *RasGAP1* provide examples of loci with promisingly narrow *F_ST_* peaks at the SNP level (Fig. 5), which may merit targeted investigation by future studies. *msn* and *RasGAP1* are also within wing QTLs and are therefore relevant to the analysis described below as well.

**Fig. 6.**
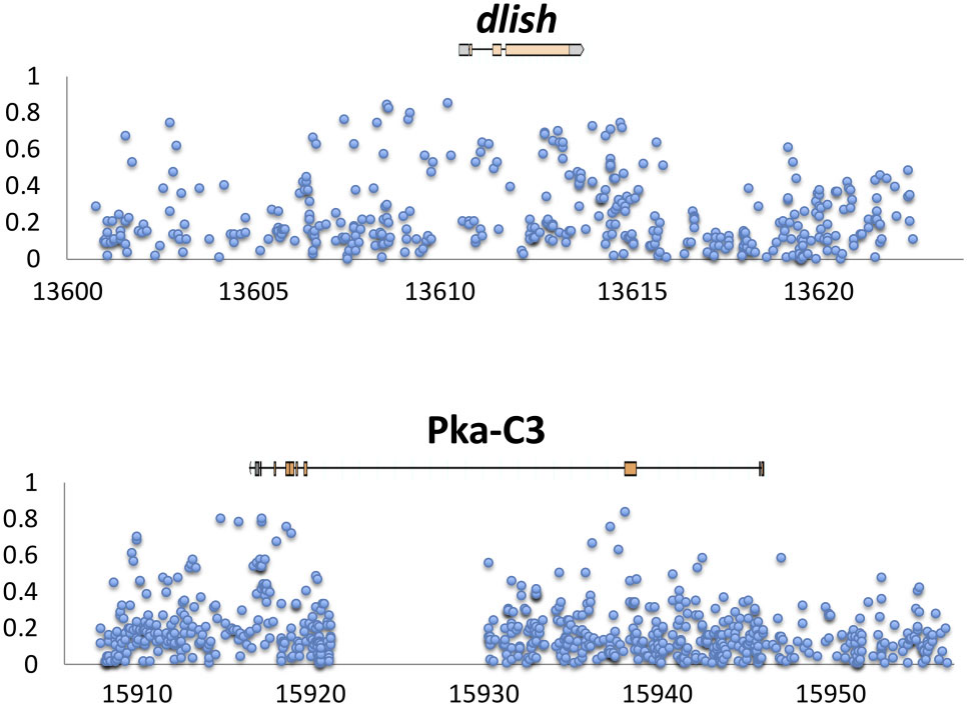
Peaks of SNP *F_ST_* center on 2 candidate genes identified for wing size evolution (*dlish* and *Pka-C3*), showing elevated genetic differentiation between Ethiopia and Zambia at these genes. Depicted above is the gene transcript, while the *x*-axis indicates kb position along the relevant chromosome arm (release 5).

Within the outlier regions for wing length, these genes included *caps* (Milán et al. 2001; Carreira et al. 2009; Jumbo-Lucioni et al. 2010; Watanabe and Riddle 2021), *dally* (Takeo et al. 2005; Carreira et al. 2009), *Dlish* (Wang et al. 2019), *Dronc* (Verghese et al. 2012), *fj* (Villano and Katz 1995), *Gbp1* (Koyama and Mirth 2016), *Pka-C3* (Dworkin and Gibson 2006; Jumbo-Lucioni et al. 2010), and *salr* (Wang et al. 2017)*. Dlish* and *Pka-C3* are examples of genes with individual SNPs having high *F_ST_* values (Fig. 6). Functional testing will be needed to establish if genetic variants found within these genes are indeed responsible for the associated phenotypes.

### GO enrichment

We conducted individual GO enrichment analysis for thorax and wing length. We used only the genes found in the outlier windows located within significant QTL regions from the 4 crosses. Full results are presented in Supplementary Tables 6 and 7. Categories with raw *P*-values below 0.001 included some that are either known or potentially involved in body and wing size. For thorax size, the top categories included: negative regulation of Ras protein signal transduction (Prober and Edgar 2002), regulation of protein polymerization (Fernández et al. 2011), and brahma complex (Krupp et al. 2005). For wing size, the top categories included: neurogenesis (Rutledge et al. 1992), ubiquitin protein ligase binding (Cornell et al. 1999), cellular amino acid catabolic process (Zinke et al. 1999), cellular response to anoxia (Heinrich et al. 2011), transmembrane transport (Bartscherer et al. 2006), and negative regulation of proteolysis (Lee et al. 2001). Some of these functional processes might underlie Ethiopia size adaptation, while others may be driven by unrelated trait evolution in this high-altitude population.

## Discussion

We employed quantitative and population genetic strategies to investigate the genetic architecture of adaptive size evolution in our highland Ethiopia population. Our bulk segregant analysis revealed that between the 4 crosses, thorax size has 12 associated QTLs with moderate-to-large effect (∼13–20%). However, between the 4 crosses wing size has 33 QTLs with small-to-large effects QTLs (∼6–27%). A greater ability to detect wing length QTLs than thorax length QTLs may reflect the greater magnitude of the population difference in this trait (Lack et al. 2016a).

One striking result was the lack of QTL overlap between crosses for either thorax or wing size. Between the 4 thorax crosses, there is no overlap. This is especially notable given that we have almost have very high power to detect QTLs with effect size of 20% (Pool 2016) and yet the QTL on chromosome arm 2L with ∼20% effect size is not present in any other cross. For wing size, there was overlap in only 12 of the 33 QTL regions and no overlap between all 4 crosses. The QTLs with the 3 largest effect sizes of over 25% are present in only one cross. The low QTL overlap between crosses could reflect persistent genetic variation at causative loci in the Ethiopian and/or Zambian populations. Given that the Ethiopian population appears to have experienced directional selection for larger size and still maintains similar genetic variance for size traits as Zambia (Lack et al. 2016b), we suggest that some favored size variants have not reached fixation in the Ethiopian population. There are multiple reasons why favored alleles might not fix, including the Ethiopian population reaching its new optimum or threshold trait value (especially if ample standing variation means that not all large alleles needed to fix), heterozygote advantage, or ongoing adaptation. Indeed, simulation and theory have shown that depending on the genetic architecture of an adaptive trait, nonfixed causative variants may be the norm (Stephan 2016; Höllinger et al. 2019; Thornton 2019; Barghi and Schlötterer 2020; Barghi et al. 2020; John and Stephan 2020).

Our conclusions of persistent variability underlying an evolved trait mirror similar results for pigmentation (Bastide et al. 2016) and for ethanol resistance (Sprengelmeyer and Pool 2021) in this same population and others, all from mapping experiments with similar design and scale. With 5 traits now examined (ethanol resistance, abdominal background color, abdominal stripe width, thorax length, and wing length), consistent patterns are starting to emerge. First, these traits each average at least a few detectable QTLs per cross, with means ranging from 3 to 8.25 (Table 1). Second, there is notably little QTL peak overlap between parallel mapping crosses involving different strains from the same populations, with QTL overlap proportions ranging from 0% to 35% (Table 1). For each of these traits, there are moderately large effect QTLs, associated with very high detection power (Pool 2016), which are not present in other crosses. Hence, at the population level, it is fair to say that each of these traits is at least moderately polygenic and involves nonfixed differences between populations. Third, moderately strong QTLs are consistently present in any given cross, with the average QTL effect size ranging from 13% to 19% (Table 1), although undetectable smaller effects may be present as well.

**Table 1.** The results of bulk QTL mapping experiments for 5 different traits.

Trait	Avg. QTLs per cross	Pairwise QTL overlap (%)	Avg. effect size (%)
Ethanol resistance	8	35	14
Thorax length	3	0	15
Wing length	8.25	36	17
Stripe width	3	8	19
Background color	5.67	18	19

All mapping used the same experimental design described in the *Materials and Methods*, aside from minor variation in the number of generations of interbreeding (15–20). Both the pigmentation stripe and pigmentation background data are from Bastide et al. (2016), while the ethanol results are from Sprengelmeyer and Pool (2021). Listed are the number of significant QTLs for each mapping population, the proportion of QTLs that overlap between parallel crosses from the same 2 populations, and the average QTL effect size across all mapping crosses.

Results compatible with the above findings have also been obtained from other experimental scenarios and from other species. For example, Barghi et al. (2019) studied the adaptation of replicate *D. simulans* populations to high temperature and found that the resulting heterogeneity in genomic responses among replicates was well-modeled by a scenario of genetic redundancy in which not all favored variants are needed to achieve an adaptive change. An experimental selection study involving desiccation resistance in *D. melanogaster* also observed considered heterogeneity among replicates (Griffin et al. 2017). Among human populations, the strongest known examples of local adaptation involve nonfixed genetic differences, including lactase persistence in northern Europeans and some African populations (e.g. Tishkoff et al. 2007; Itan et al. 2009), high-altitude adaptation in Andeans/Ethiopians/Tibetans (e.g. Bigham et al. 2009; Yi et al. 2010; Huerta-Sánchez et al. 2013), and enhanced diving ability in the Bajau (Ilardo et al. 2018). Likewise, shifts in natural stickleback populations have also involved nonfixed genetic changes, with pleiotropy suggested to play a role (Rogers et al. 2012). Similarly, pleiotropic effects of insecticide resistance variants may help explain why they are often in natural populations of *D. melanogaster* and other species (e.g. Catania et al. 2004; Clarkson et al. 2021). We also note that a population genetic analysis of African populations of *D. melanogaster* found ample evidence for incomplete sweeps (Vy et al. 2017).

Polygenic adaptation may have diverse outcomes, depending in part on the number of segregating variants at the onset of selection that affect a trait, as well as the magnitudes of their effect on the trait relative to the shift in trait optimum. While each of the traits summarized above might be described as “polygenic,” it is worth considering the type of polygenic adaptation that these mapping studies imply. The persistently variable genetic basis of these evolved traits may suggest a scenario of abundant standing genetic variation prior to selection for each of these traits. In light of the consistent presence of moderately strong QTLs for these traits, such standing variation may have included relatively large effect loci, which would experience relatively stronger directional selection during the trait’s evolution. An abundance of standing variation is consistent with the large population size and high genetic diversity of this species (e.g. Sprengelmeyer et al. 2020). Further studies will be needed to quantify the models of polygenic adaptation that experiments such as ours indicate, and to assess whether such persistent variability is a widespread outcome of trait evolution not only in this species but also across the tree of life.

## Data availability

All raw sequence data have been deposited in the NIH Short Read Archive, with accession numbers given in Supplementary Table 1. The scripts used for SIBSAM can be found at: http://github.com/JohnEPool/SIBSAM1; last accessed January 5, 2022.

Supplemental material is available at *G3* online.

## Supplementary Material

jkab454_Supplementary_Table_S1

jkab454_Supplementary_Table_S2

jkab454_Supplementary_Table_S3

jkab454_Supplementary_Table_S4

jkab454_Supplementary_Table_S5

jkab454_Supplementary_Table_S6

jkab454_Supplementary_Table_S7

## References

[jkab454-B2] Barghi N , Hermisson J, Schlötterer C. Polygenic adaptation: a unifying framework to understand positive selection. Nat Rev Genet. 2020;21(12):769–781. 32601318 10.1038/s41576-020-0250-z

[jkab454-B3] Barghi N , Tobler R, Nolte V, Jakšić AM, Mallard F, Otte KA, Dolezal M, Taus T, Kofler R, Schlötterer C, et al Genetic redundancy fuels polygenic adaptation in *Drosophila*. PLoS Biol. 2019;17(2):e3000128. 30716062 10.1371/journal.pbio.3000128 PMC6375663

[jkab454-B4] Bartscherer K , Pelte N, Ingelfinger D, Boutros M. Secretion of Wnt ligands requires Evi, a conserved transmembrane protein. Cell. 2006;125(3):523–533. 16678096 10.1016/j.cell.2006.04.009

[jkab454-B6] Bastide H , Lange JD, Lack JB, Yassin A, Pool JE. A variable genetic architecture of melanic evolution in *Drosophila melanogaster*. Genetics. 2016;204(3):1307–1319. 27638419 10.1534/genetics.116.192492 PMC5105859

[jkab454-B7] Bejarano F , Luque CM, Herranz H, Sorrosal G, Rafel N, Pham TT, Milán M. A gain-of-function suppressor screen for genes involved in dorsal–ventral boundary formation in the *Drosophila* wing. Genetics. 2008;178(1):307–323. 18202376 10.1534/genetics.107.081869 PMC2206080

[jkab454-B8] Bigham AW , Mao X, Mei R, Brutsaert T, Wilson MJ, Julian CG, Parra EJ, Akey JM, Moore LG, Shriver MD, et al Identifying positive selection candidate loci for high-altitude adaptation in Andean populations. Hum Genomics. 2009;4(2):79–12. 20038496 10.1186/1479-7364-4-2-79 PMC2857381

[jkab454-B10] Calboli FCF , Kennington WJ, Partridge L. QTL mapping reveals a striking coincidence in the positions of genomic regions associated with adaptive variation in body size in parallel clines of *Drosophila melanogaster* on different continents. Evolution. 2003;57(11):2653–2658. 14686541 10.1111/j.0014-3820.2003.tb01509.x

[jkab454-B11] Carreira VP , Mensch J, Fanara JJ. Body size in *Drosophila*: genetic architecture, allometries and sexual dimorphism. Heredity (Edinb). 2009;102(3):246–256. 19018274 10.1038/hdy.2008.117

[jkab454-B12] Carroll SB. Evo-devo and an expanding evolutionary synthesis: a genetic theory of morphological evolution. Cell. 2008;134(1):25–36. 18614008 10.1016/j.cell.2008.06.030

[jkab454-B13] Catania F , Kauer MO, Daborn PJ, Yen JL, Ffrench‐Constant RH, Schlötterer C. World‐wide survey of an Accord insertion and its association with DDT resistance in *Drosophila melanogaster*. Mol Ecol. 2004;13(8):2491–2504. 15245421 10.1111/j.1365-294X.2004.02263.x

[jkab454-B14] Chen Y , Lee SF, Blanc E, Reuter C, Wertheim B, Martinez-Diaz P, Hoffmann AA, Partridge L. Genome-wide transcription analysis of clinal genetic variation in *Drosophila*. PLoS One. 2012;7(4):e34620. 22514645 10.1371/journal.pone.0034620 PMC3326059

[jkab454-B15] Clarkson CS , Miles A, Harding NJ, O'Reilly AO, Weetman D, Kwiatkowski D, Donnelly MJ; Anopheles gambiae 1000 Genomes Consortium. The genetic architecture of target‐site resistance to pyrethroid insecticides in the African malaria vectors *Anopheles gambiae* and *Anopheles coluzzii*. Mol Ecol. 2021;30(21):5303–5317. 33590926 10.1111/mec.15845 PMC9019111

[jkab454-B16] Collinge JE , Hoffmann AA, McKechnie SW. Altitudinal patterns for latitudinally varying traits and polymorphic markers in *Drosophila melanogaster* from eastern Australia. J Evolution Biol. 2006;19(2):473–482. 10.1111/j.1420-9101.2005.01016.x 16599923

[jkab454-B17] Colosimo PF , Hosemann KE, Balabhadra S, Villarreal G, Dickson M, Grimwood J, Schmutz J, Myers RM, Schluter D, Kingsley DM. Widespread parallel evolution in sticklebacks by repeated fixation of ectodysplasin alleles. Science. 2005;307(5717):1928–1933. 15790847 10.1126/science.1107239

[jkab454-B18] Cornell M , Evans D. A P, Mann R, Fostier M, Flasza M, Monthatong M, Artavanis-Tsakonas S, Baron M. The Drosophila melanogaster suppressor of deltex gene, a regulator of the Notch receptor signaling pathway, is an E3 class ubiquitin ligase. Genetics. 1999;152(2):567–576. 10353900 10.1093/genetics/152.2.567 PMC1460625

[jkab454-B19] David JR , Bocquet C, de Scheemaeker-Louis M. Genetic latitudinal adaptation of *Drosophila melanogaster*: new discriminative biometrical traits between European and equatorial African populations. Genet Res. 1977;30(3):247–255.

[jkab454-B21] Dillon ME , Frazier MR. *Drosophila melanogaster* locomotion in cold thin air. J Exp Biol. 2006;209(Pt 2):364–371. 16391358 10.1242/jeb.01999

[jkab454-B22] Dworkin I , Gibson G. Epidermal growth factor receptor and transforming growth factor-β signaling contributes to variation for wing shape in *Drosophila melanogaster*. Genetics. 2006;173(3):1417–1431. 16648592 10.1534/genetics.105.053868 PMC1526698

[jkab454-B23] Edgar BA , Orr-Weaver TL. Endoreplication cell cycles: more for less. Cell. 2001;105(3):297–306. 11348589 10.1016/s0092-8674(01)00334-8

[jkab454-B24] Fabian DK , Lack JB, Mathur V, Schlötterer C, Schmidt PS, Pool JE, Flatt T. Spatially varying selection shapes life history clines among populations of *Drosophila melanogaster* from sub-Saharan Africa. J Evol Biol. 2015;28(4):826–840. 25704153 10.1111/jeb.12607 PMC4405473

[jkab454-B25] Fernández BG , Gaspar P, Brás-Pereira C, Jezowska B, Rebelo SR, Janody F. Actin-Capping Protein and the Hippo pathway regulate F-actin and tissue growth in *Drosophila*. Development. 2011;138(11):2337–2346. 21525075 10.1242/dev.063545

[jkab454-B26] Frazier MR , Harrison JF, Kirkton SD, Roberts SP. Cold rearing improves cold-flight performance in *Drosophila* via changes in wing morphology. J Exp Biol. 2008;211(Pt 13):2116–2122. 18552301 10.1242/jeb.019422

[jkab454-B27] Gilchrist AS , Partridge L. A comparison of the genetic basis of wing size divergence in three parallel body size clines of *Drosophila melanogaster*. Genetics. 1999;153(4):1775–1787. 10581284 10.1093/genetics/153.4.1775 PMC1460863

[jkab454-B28] Griffin PC , Hangartner SB, Fournier-Level A, Hoffmann AA. Genomic trajectories to desiccation resistance: convergence and divergence among replicate selected *Drosophila* lines. Genetics. 2017;205(2):871–890. 28007884 10.1534/genetics.116.187104 PMC5289857

[jkab454-B29] Heinrich EC , Farzin M, Klok CJ, Harrison JF. The effect of developmental stage on the sensitivity of cell and body size to hypoxia in *Drosophila melanogaster*. J Exp Biol. 2011;214(Pt 9):1419–1427. 21490250 10.1242/jeb.051904 PMC3076073

[jkab454-B31] Hoffmann AA , Ratna E, Sgro CM, Barton M, Blacket M, Hallas R, De Garis S, Weeks AR. Antagonistic selection between adult thorax and wing size in field released *Drosophila melanogaster* independent of thermal conditions. J Evol Biol. 2007;20(6):2219–2227. 17887974 10.1111/j.1420-9101.2007.01422.x

[jkab454-B32] Höllinger I , Pennings PS, Hermisson J. Polygenic adaptation: from sweeps to subtle frequency shifts. PLoS Genet. 2019;15(3):e1008035. 30893299 10.1371/journal.pgen.1008035 PMC6443195

[jkab454-B99] Houle D , Mezey J, Galpern P, Carter A. Automated measurement of Drosophila wings. BMC Evol Biol. 2003;3(1):1–3. 14670094 10.1186/1471-2148-3-25 PMC317280

[jkab454-B33] Huerta-Sánchez E , Degiorgio M, Pagani L, Tarekegn A, Ekong R, Antao T, Cardona A, Montgomery HE, Cavalleri GL, Robbins PA, et al Genetic signatures reveal high-altitude adaptation in a set of Ethiopian populations. Mol Biol Evol. 2013;30(8):1877–1888. 23666210 10.1093/molbev/mst089 PMC3708501

[jkab454-B100] Ilardo MA , Moltke I, Korneliussen TS, Cheng J, Stern AJ, Racimo F, de Barros Damgaard P, Sikora M, Seguin-Orlando A, Rasmussen S et al Physiological and genetic adaptations to diving in sea nomads. Cell. 2018;173(3):569–580. 29677510 10.1016/j.cell.2018.03.054

[jkab454-B34] Itan Y , Powell A, Beaumont MA, Burger J, Thomas MG. The origins of lactase persistence in Europe. PLoS Comput Biol. 2009;5(8):e1000491. 19714206 10.1371/journal.pcbi.1000491 PMC2722739

[jkab454-B36] John S , Stephan W. Important role of genetic drift in rapid polygenic adaptation. Ecol Evol. 2020;10(3):1278–1287. 32076513 10.1002/ece3.5981 PMC7029068

[jkab454-B37] Jumbo-Lucioni P , Ayroles JF, Chambers MM, Jordan KW, Leips J, Mackay TF, De Luca M. Systems genetics analysis of body weight and energy metabolism traits in *Drosophila melanogaster*. BMC Genomics. 2010;11(1):297. 20459830 10.1186/1471-2164-11-297 PMC2880307

[jkab454-B38] Kadrmas JL , Smith MA, Clark KA, Pronovost SM, Muster N, Yates JR III, Beckerle MC. The integrin effector PINCH regulates JNK activity and epithelial migration in concert with Ras suppressor 1. J Cell Biol. 2004;167(6):1019–1024. 15596544 10.1083/jcb.200408090 PMC2034365

[jkab454-B39] Klepsatel P , Gáliková M, Maio ND, Huber CD, Schlötterer C, Flatt T. Variation in thermal performance and reaction norms among populations of *Drosophila melanogaster*. Evolution. 2013;67(12):3573–3587. 24299409 10.1111/evo.12221

[jkab454-B40] Klepsatel P , Gáliková M, Huber CD, Flatt T. Similarities and differences in altitudinal versus latitudinal variation for morphological traits in *Drosophila melanogaster*. Evolution. 2014;68(5):1385–1398. 24410363 10.1111/evo.12351

[jkab454-B41] Kofler R , Pandey RV, Schlötterer C. PoPoolation2: identifying differentiation between populations using sequencing of pooled DNA samples (Pool-Seq). Bioinformatics. 2011;27(24):3435–3436. 22025480 10.1093/bioinformatics/btr589 PMC3232374

[jkab454-B42] Koyama T , Mirth CK. Growth-blocking peptides as nutrition-sensitive signals for insulin secretion and body size regulation. PLoS Biol. 2016;14(2):e1002392. 26928023 10.1371/journal.pbio.1002392 PMC4771208

[jkab454-B43] Krupp JJ , Yaich LE, Wessells RJ, Bodmer R. Identification of genetic loci that interact with cut during *Drosophila* wing-margin development. Genetics. 2005;170(4):1775–1795. 15956666 10.1534/genetics.105.043125 PMC1449764

[jkab454-B44] Lack JB , Cardeno CM, Crepeau MW, Taylor W, Corbett-Detig RB, Stevens KA, Langley CH, Pool JE. The *Drosophila* Genome Nexus: a population genomic resource of 623 *Drosophila melanogaster* genomes, including 197 from a single ancestral range population. Genetics. 2015;199(4):1229–1241. 25631317 10.1534/genetics.115.174664 PMC4391556

[jkab454-B45] Lack JB , Monette MJ, Johanning EJ, Sprengelmeyer QD, Pool JE. Decanalization of wing development accompanied the evolution of large wings in high-altitude *Drosophila*. Proc Natl Acad Sci U S A. 2016a;113(4):1014–1019. 26755605 10.1073/pnas.1515964113 PMC4743785

[jkab454-B46] Lack JB , Yassin A, Sprengelmeyer QD, Johanning EJ, David JR, Pool JE. Life history evolution and cellular mechanisms associated with increased size in high‐altitude *Drosophila*. Ecol Evol. 2016b;6(16):5893–5906. 27547363 10.1002/ece3.2327 PMC4983600

[jkab454-B47] Lack JB , Lange JD, Tang AD, Corbett-Detig RB, Pool JE. A thousand fly genomes: an expanded *Drosophila* Genome Nexus. Mol Biol Evol. 2016c;33(12):3308–3313. 27687565 10.1093/molbev/msw195 PMC5100052

[jkab454-B48] Lafuente E , Duneau D, Beldade P. Genetic basis of thermal plasticity variation in *Drosophila melanogaster* body size. PLoS Genet. 2018;14(9):e1007686. 30256798 10.1371/journal.pgen.1007686 PMC6175520

[jkab454-B49] Lange JD , Pool JE. A haplotype method detects diverse scenarios of local adaptation from genomic sequence variation. Mol Ecol. 2016;25(13):3081–3100. 27135633 10.1111/mec.13671 PMC4931985

[jkab454-B50] Lee JR , Urban S, Garvey CF, Freeman M. Regulated intracellular ligand transport and proteolysis control EGF signal activation in *Drosophila*. Cell. 2001;107(2):161–171. 11672524 10.1016/s0092-8674(01)00526-8

[jkab454-B51] Lee SF , Chen Y, Varan AK, Wee CW, Rako L, Axford JK, Good RT, Blacket MJ, Reuter C, Partridge L, et al Molecular basis of adaptive shift in body size in *Drosophila melanogaster*: functional and sequence analyses of the Dca gene. Mol Biol Evol. 2011;28(8):2393–2402. 21393605 10.1093/molbev/msr064

[jkab454-B52] Li H , Durbin R. Fast and accurate short read alignment with Burrows–Wheeler transform. Bioinformatics. 2009;25(14):1754–1760. 19451168 10.1093/bioinformatics/btp324 PMC2705234

[jkab454-B53] Liao B-Y , Weng M-P, Zhang J. Contrasting genetic paths to morphological and physiological evolution. Proc Natl Acad Sci U S A. 2010;107(16):7353–7358. 20368429 10.1073/pnas.0910339107 PMC2867737

[jkab454-B54] Linnen CR , Kingsley EP, Jensen JD, Hoekstra HE. On the origin and spread of an adaptive allele in deer mice. Science. 2009;325(5944):1095–1098. 19713521 10.1126/science.1175826 PMC2736094

[jkab454-B55] Liu Y , Wang W, Shui G, Huang X. CDP-Diacylglycerol Synthetase coordinates cell growth and fat storage through phosphatidylinositol metabolism and the insulin pathway. PLoS Genet. 2014;10(3):e1004172. 24603715 10.1371/journal.pgen.1004172 PMC3945474

[jkab454-B56] Louis J , David J, Rouault J, Capy P. Altitudinal variations of Afro-tropical D. melanogaster populations. Drosoph Inf Serv. 1982;58:100–101.

[jkab454-B57] Lunter G , Goodson M. Stampy: a statistical algorithm for sensitive and fast mapping of Illumina sequence reads. Genome Res. 2011;21(6):936–939. 20980556 10.1101/gr.111120.110 PMC3106326

[jkab454-B58] McCabe J , Partridge L. An interaction between environmental temperature and genetic variation for body size for the fitness of adult female *Drosophila melanogaster*. Evolution. 1997;51(4):1164–1174. 28565476 10.1111/j.1558-5646.1997.tb03964.x

[jkab454-B59] McKenna A , Hanna M, Banks E, Sivachenko A, Cibulskis K, Kernytsky A, Garimella K, Altshuler D, Gabriel S, Daly M, et al The Genome Analysis Toolkit: a MapReduce framework for analyzing next-generation DNA sequencing data. Genome Res. 2010;20(9):1297–1303. 20644199 10.1101/gr.107524.110 PMC2928508

[jkab454-B60] Milán M , Weihe U, Pérez L, Cohen SM. The LRR proteins capricious and Tartan mediate cell interactions during DV boundary formation in the *Drosophila* wing. Cell. 2001;106(6):785–794. 11572783 10.1016/s0092-8674(01)00489-5

[jkab454-B61] Miller CT , Glazer AM, Summers BR, Blackman BK, Norman AR, Shapiro MD, Cole BL, Peichel CL, Schluter D, Kingsley DM. Modular skeletal evolution in sticklebacks is controlled by additive and clustered quantitative trait loci. Genetics. 2014;197(1):405–420. 24652999 10.1534/genetics.114.162420 PMC4012497

[jkab454-B62] Nagaraj R , Pickup AT, Howes R, Moses K, Freeman M, Banerjee U. Role of the EGF receptor pathway in growth and patterning of the *Drosophila* wing through the regulation of vestigial. Development. 1999;126(5):975–985. 9927598 10.1242/dev.126.5.975

[jkab454-B63] Norry FM , Bubliy OA, Loeschcke V. Developmental time, body size and wing loading in *Drosophila buzzatii* from lowland and highland populations in Argentina. Hereditas. 2001;135(1):35–40. 12035612 10.1111/j.1601-5223.2001.t01-1-00035.x

[jkab454-B97] Partridge L , French V. Thermal evolution of ectotherm body size: Why get big in the cold. 1996; In Johnston I., Bennett A. (Eds.), Animals and Temperature: Phenotypic and Evolutionary Adaptation, p. 265–292. Cambridge, UK: Cambridge University Press.

[jkab454-B64] Pennings PS , Hermisson J. Soft Sweeps III: the signature of positive selection from recurrent mutation. PLOS Genet. 2006;2(12):e186. 17173482 10.1371/journal.pgen.0020186 PMC1698945

[jkab454-B65] Pitchers W , Pool JE, Dworkin I. Altitudinal clinal variation in wing size and shape in African *Drosophila melanogaster*: one cline or many? Evolution. 2013;67(2):438–452. 23356616 10.1111/j.1558-5646.2012.01774.x PMC3786396

[jkab454-B66] Pool JE , Corbett-Detig RB, Sugino RP, Stevens KA, Cardeno CM, Crepeau MW, Duchen P, Emerson JJ, Saelao P, Begun DJ, et al Population genomics of sub-Saharan *Drosophila melanogaster*: African diversity and non-African admixture. PLoS Genet. 2012;8(12):e1003080. 23284287 10.1371/journal.pgen.1003080 PMC3527209

[jkab454-B67] Pool JE. The mosaic ancestry of the *Drosophila* genetic reference panel and the D. melanogaster reference genome reveals a network of epistatic fitness interactions. Mol Biol Evol. 2015;32(12):3236–3251. 26354524 10.1093/molbev/msv194 PMC4652625

[jkab454-B68] Pool JE. Genetic mapping by bulk segregant analysis in *Drosophila*: experimental design and simulation-based inference. Genetics. 2016;204(3):1295–1306. 27655945 10.1534/genetics.116.192484 PMC5105858

[jkab454-B70] Pritchard JK , Di Rienzo A. Adaptation—not by sweeps alone. Nat Rev Genet. 2010;11(10):665–667. 20838407 10.1038/nrg2880 PMC4652788

[jkab454-B71] Prober DA , Edgar BA. Interactions between Ras1, dMyc, and dPI3K signaling in the developing *Drosophila* wing. Genes Dev. 2002;16(17):2286–2299. 12208851 10.1101/gad.991102 PMC186666

[jkab454-B72] Reeve MW , Fowler K, Partridge L. Increased body size confers greater fitness at lower experimental temperature in male *Drosophila melanogaster*. J Evol Biol. 2000;13(5):836–844.

[jkab454-B73] Reiling JH , Hafen E. The hypoxia-induced paralogs *Scylla* and *Charybdis* inhibit growth by down-regulating S6K activity upstream of TSC in *Drosophila*. Genes Dev. 2004;18(23):2879–2892. 15545626 10.1101/gad.322704 PMC534649

[jkab454-B74] Rockman MV. The QTN Program and the alleles that matter for evolution: all that’s gold does not glitter. Evolution. 2012;66(1):1–17. 22220860 10.1111/j.1558-5646.2011.01486.x PMC3386609

[jkab454-B75] Rogers SM , Tamkee P, Summers B, Balabahadra S, Marks M, Kingsley DM, Schluter D. Genetic signature of adaptive peak shift in threespine stickleback. Evolution. 2012;66(8):2439–2450. 22834743 10.1111/j.1558-5646.2012.01622.x PMC4433314

[jkab454-B76] Rutledge BJ , Zhang K, Bier E, Jan YN, Perrimon N. The *Drosophila spitz* gene encodes a putative EGF-like growth factor involved in dorsal-ventral axis formation and neurogenesis. Genes Dev. 1992;6(8):1503–1517. 1644292 10.1101/gad.6.8.1503

[jkab454-B77] Smith AV , Orr-Weaver TL. The regulation of the cell cycle during *Drosophila* embryogenesis: the transition to polyteny. Development. 1991;112(4):997–1008. 1935703 10.1242/dev.112.4.997

[jkab454-B78] Sprengelmeyer QD , Mansourian S, Lange JD, Matute DR, Cooper BS, Jirle EV, Stensmyr MC, Pool JE. Recurrent collection of *Drosophila melanogaster* from wild African environments and genomic insights into species history. Mol Biol Evol. 2020;37(3):627–638. 31730190 10.1093/molbev/msz271 PMC7038662

[jkab454-B79] Sprengelmeyer QD , Pool JE. Ethanol resistance in *Drosophila melanogaster* has increased in parallel cold-adapted populations and shows a variable genetic architecture within and between populations. Ecol Evol. 2021;11(21):15364–15376. 34765183 10.1002/ece3.8228 PMC8571616

[jkab454-B80] Stalker HD , Carson HL. An altitudinal transect of *Drosophila robusta* Sturtevant. Evolution. 1948;2(4):295–305. 18104587 10.1111/j.1558-5646.1948.tb02747.x

[jkab454-B81] Stephan W. Signatures of positive selection: from selective sweeps at individual loci to subtle allele frequency changes in polygenic adaptation. Mol Ecol. 2016;25(1):79–88. 26108992 10.1111/mec.13288

[jkab454-B82] Takeo S , Akiyama T, Firkus C, Aigaki T, Nakato H. Expression of a secreted form of Dally, a *Drosophila* glypican, induces overgrowth phenotype by affecting action range of Hedgehog. Dev Biol. 2005;284(1):204–218. 15963974 10.1016/j.ydbio.2005.05.014

[jkab454-B83] Tishkoff SA , Reed FA, Ranciaro A, Voight BF, Babbitt CC, Silverman JS, Powell K, Mortensen HM, Hirbo JB, Osman M, et al Convergent adaptation of human lactase persistence in Africa and Europe. Nat Genet. 2007;39(1):31–40. 17159977 10.1038/ng1946 PMC2672153

[jkab454-B84] Thornton KR. Polygenic Adaptation to an environmental shift: temporal dynamics of variation under gaussian stabilizing selection and additive effects on a single trait. Genetics. 2019;213(4):1513–1530. 31653678 10.1534/genetics.119.302662 PMC6893385

[jkab454-B85] Thumm M , Kadowaki T. The loss of *Drosophila* APG4/AUT2 function modifies the phenotypes of cut and Notch signaling pathway mutants. Mol Genet Genomics. 2001;266(4):657–663. 11810238 10.1007/s004380100585

[jkab454-B86] Turner TL , Stewart AD, Fields AT, Rice WR, Tarone AM. Population-based resequencing of experimentally evolved populations reveals the genetic basis of body size variation in *Drosophila melanogaster*. PLoS Genet. 2011;7(3):e1001336. 21437274 10.1371/journal.pgen.1001336 PMC3060078

[jkab454-B98] Turelli M , Hoffmann AA. Cytoplasmic incompatibility in Drosophila simulans: dynamics and parameter estimates from natural populations. Genetics. 1995;140(4):1319–1338. 7498773 10.1093/genetics/140.4.1319 PMC1206697

[jkab454-B87] van’t Hof AE , Edmonds N, Dalíková M, Marec F, Saccheri IJ. Industrial melanism in British peppered moths has a singular and recent mutational origin. Science. 2011;332(6032):958–960. 21493823 10.1126/science.1203043

[jkab454-B88] Verghese S , Bedi S, Kango-Singh M. Hippo signalling controls Dronc activity to regulate organ size in *Drosophila*. Cell Death Differ. 2012;19(10):1664–1676. 22555454 10.1038/cdd.2012.48 PMC3438497

[jkab454-B89] Villano JL , Katz FN. Four-jointed is required for intermediate growth in the proximal-distal axis in *Drosophila*. Development. 1995;121(9):2767–2777. 7555705 10.1242/dev.121.9.2767

[jkab454-B90] Vy HMT , Won YJ, Kim Y. Multiple modes of positive selection shaping the patterns of incomplete selective sweeps over African populations of *Drosophila melanogaster*. Mol Biol Evol. 2017;34(11):2792–2807. 28981697 10.1093/molbev/msx207

[jkab454-B91] Wang D , Li J, Liu S, Zhou H, Zhang L, Shi W, Shen J. Spalt is functionally conserved in *Locusta* and *Drosophila* to promote wing growth. Sci Rep. 2017;7(1):44393. 28300136 10.1038/srep44393 PMC5353606

[jkab454-B92] Wang X , Zhang Y, Blair SS. Fat-regulated adaptor protein Dlish binds the growth suppressor Expanded and controls its stability and ubiquitination. Proc Natl Acad Sci U S A. 2019;116(4):1319–1324. 30606799 10.1073/pnas.1811891116 PMC6347691

[jkab454-B93] Watanabe LP , Riddle NC. GWAS reveal a role for the central nervous system in regulating weight and weight change in response to exercise. Sci Rep. 2021;11(1):1–13. 33664357 10.1038/s41598-021-84534-w PMC7933348

[jkab454-B94] Yeaman S , Whitlock MC. The genetic architecture of adaptation under migration–selection balance. Evolution. 2011;65(7):1897–1911. 21729046 10.1111/j.1558-5646.2011.01269.x

[jkab454-B95] Yi X , Liang Y, Huerta-Sanchez E, Jin X, Cuo ZXP, Pool JE, Xu X, Jiang H, Vinckenbosch N, Korneliussen TS, et al Sequencing of 50 human exomes reveals adaptation to high altitude. Science. 2010;329(5987):75–78. 20595611 10.1126/science.1190371 PMC3711608

[jkab454-B96] Zinke I , Kirchner C, Chao LC, Tetzlaff MT, Pankratz MJ. Suppression of food intake and growth by amino acids in *Drosophila*: the role of pumpless, a fat body expressed gene with homology to vertebrate glycine cleavage system. Development. 1999;126(23):5275–5284. 10556053 10.1242/dev.126.23.5275

